# Identification of Transcription Factor AML-1 Binding Site Upstream of Human Cytomegalovirus *UL111A* Gene

**DOI:** 10.1371/journal.pone.0117773

**Published:** 2015-02-06

**Authors:** Xiaoqun Zheng, Yan Gao, Qi Zhang, Yanqing Liu, Ying Peng, Miao Fu, Yanhong Ji

**Affiliations:** 1 Department of Immunology and Pathogenic Biology, School of Medicine, Xi’an Jiaotong University, Xi’an, Shaanxi, China; 2 Department of Laboratory Medicine, the Second Affiliated Hospital & Yuying Children’s Hospital of Wenzhou Medical University, Wenzhou, Zhejiang, China; 3 School of Laboratory Medicine, Wenzhou Medical University, Wenzhou, Zhejiang, China; 4 Key Laboratory of Laboratory Medicine, Education Ministry of China, Wenzhou, Zhejiang, China; 5 The First People’s Hospital of Changde, Changde, Hunan, China; 6 Jinhua Municipal Central Hospital, Jinhua, Zhejiang, China; Fudan University, CHINA

## Abstract

Human cytomegalovirus (HCMV) interleukin-10 (hcmvIL-10), encoded by HCMV *UL111A* gene, is a homolog of human IL-10. It exerts immunomodulatory effects that allow HCMV to evade host defense mechanisms. However, the exact mechanism underlying the regulation of hcmvIL-10 expression is not well understood. The transcription factor acute myeloid leukemia 1 (AML-1) plays an important role in the regulation of various genes involved in the differentiation of hematopoietic lineages. A putative AML-1 binding site is present within the upstream regulatory region (URR) of *UL111A* gene. To provide evidence that AML-1 is involved in regulating *UL111A* gene expression, we examined the interaction of AML-1 with the URR of *UL111A* in HCMV-infected human monocytic THP-1 cells using a chromatin immunoprecipitation assay. HcmvIL-10 transcription was detected in differentiated THP-1 cells, but not in undifferentiated ones. Furthermore, the URR of *UL111A* showed a higher intensity of AML-1 binding, a higher level of histone H3 acetyl-K9, but a lower level of histone H3 dimethyl-K9 in differentiated THP-1 cells than undifferentiated cells. Down-regulation of AML1 by RNA interference decreased the expression of the *UL111A* gene. Our results suggest that AML-1 may contribute to the epigenetic regulation of *UL111A* gene via histone modification in HCMV-infected differentiated THP-1 cells. This finding could be useful for the development of new anti-viral therapies.

## Introduction

Human cytomegalovirus (HCMV) encoding for more than 227 proteins, a member of the β-herpesvirus subfamily, has infected the majority of the world’s population as a consequence of its highly restricted host range [1,2]. Primary HCMV infection in healthy individuals is often asymptomatic, because HCMV can establish and maintain a lifelong latent infection in myeloid progenitor cells [3–5]. However, upon HCMV reactivation from latency, it can cause serious and even life-threatening diseases including retinitis, pneumonitis, hepatitis, and gastrointestinal disease in newborns and immunocompromised individuals, such as AIDS patients and allograft recipients [6,7]. One of the strategies that HCMV has developed to evade detection by the host immune system is to encode homologs of cytokines, chemokines, and their receptors [8–11]. Those properties, shared by all herpesviruses, may promote viral infection by manipulating and evading the host immune defense mechanisms.

HCMV interleukin-10 (hcmvIL-10), which consists of 175 amino acids encoded by HCMV *UL111A* gene, has been identified as a human IL-10 (hIL-10) homolog. It shares a 27% amino acid sequence identity with hIL-10, and has similar functions, which exerts a broad range of immunosuppressive effects [12,13]. HcmvIL-10 has been reported to inhibit dendritic cell (DC) maturation by reducing MHC class ΙΙ expression, and further increase apoptosis associated with DC maturation by blocking the upregulation of the anti-apoptotic, long isoform of cellular FLICE-like inhibitory protein [14–16]. Recently, M2 macrophages were demonstrated to possess increased permissiveness of HCMV productive infection, thus raising the intriguing possibility that hcmvIL-10 might mediate the polarization of monocytes to an M2c phenotype to enhance subsequent HCMV infection [17]. Taken together, these data suggest that hcmvIL-10 contributes significantly to the immune evasion strategies of HCMV. However, the exact mechanism underlying the regulation of *UL111A* gene expression is still not well understood.

Acute myeloid leukemia 1 (AML-1), a myeloid transcription factor, plays an important role in the regulation of various genes involved in the differentiation of hematopoietic lineages [18,19]. A putative AML-1 binding site is located within the upstream regulatory region (URR) of the *UL111A* gene. However, it is not yet clear whether AML-1 is involved in controlling the expression of *UL111A*.

In this study, we aimed to elucidate the role of myeloid transcription factors in regulating *UL111A* gene expression. For this purpose, we performed chromatin immunoprecipitation (ChIP) assays to measure binding of transcription factors to the URR of *UL111A*. Furthermore, the presence of histone H3 modifications was investigated in the transcription factor binding sites of the *UL111A* URR. We analyzed the DNA methylation pattern of the *UL111A* URR in both HCMV-infected differentiated and undifferentiated THP-1 cells using the bisulfite modification method, and further examined how shifts in the HCMV URR methylation may affect viral gene expression and replication. Our findings suggest that AML-1 may contribute to the epigenetic activation of *UL111A* via histone modification in HCMV-infected differentiated THP-1 cells.

## Materials and Methods

### Cells and viruses

HEL (human embryonic lung fibroblast) cells were obtained from the Kunming Institute of Zoology of the Chinese Academy of Sciences and cultured in Dulbecco’s modified Eagle’s medium (DMEM, Gibco, USA) supplemented with 10% fetal bovine serum (FBS, Gibco), 2 mM L-glutamine, and 100 U/ml penicillin and 100 μg/ml streptomycin. THP-1 cells (a human acute monocytic leukemia cell line) were obtained from the cell bank of the Shanghai Institute, and maintained in RPMI-1640 medium (Gibco) with 10% FBS, 100 U/ml penicillin and 100 μg/ml streptomycin. All cells were cultured at 37°C in a 5% CO_2_ humidified atmosphere. Towne strain of HCMV (HCMV-Towne), obtained from American Type Culture Collection (ATCC), was a gift from Dr. Shiqiang Shang (Zhejiang University).

### Virus infection

HEL cells were used for virus production. HEL cells were typically infected at approximately 50~70% confluence with purified HCMV-Towne at a multiplicity of infection (MOI) of 5. As soon as cytopathic effects were evident, the culture supernatant containing virus was harvested and centrifuged at 4000× *g* for 5 min at 4°C. Virus stocks were stored at −80°C for future use. The supernatants from the infected HEL cells were assayed for infectivity, and viral titers were determined by plaque assay. For latency studies, THP-1 cells (1×10^6^/ml) were infected with HCMV-Towne at an MOI of 5 in 6-well plates. At 24 h post-infection, the medium was changed and the infection was allowed to proceed for 10 days, during which half of the medium was changed every 2 days. At each assay point, cells remaining in suspension were harvested and analyzed. For reactivation studies, macrophage differentiation of THP-1 cells was achieved by treating the infected cells with 100 ng/ml phorbol 12-myristate 13-acetate (PMA, Beyotime, China) for 24 h. PMA-treated cells became adherent and acquired a spindle-like morphology. The infection was allowed to proceed for 10 days, with changes of fresh medium containing PMA every 2 days. Cells and supernatants were collected separately at the indicated day post infection (dpi), and then analyzed. Viral genomes were quantified by real-time quantitative PCR (qPCR). The data presented are the averages from three independent experiments.

### Viral DNA extraction and quantitative analysis by qPCR

Infected cells were harvested, washed twice with phosphate-buffered saline (PBS), and pelleted by low-speed centrifugation. Total DNA was extracted from the cells using a QIAamp DNA Mini Kit (QIAGEN, Germany) according to the manufacturer’s instructions. The DNA concentration was determined by a NanoDrop 2000 UV-vis spectrophotometer (Thermo Scientific, USA). Subsequently, a qPCR assay was performed with primers for the viral immediate-early 1(IE1) gene to determine the levels of viral DNA loads using an ABI 7500 Real Time PCR System (Applied Biosciences, USA).

### RNA extraction and RT-PCR analysis

Total RNA was isolated using Trizol reagent (Invitrogen, USA) according to the manufacturer’s recommendations and quantified using a spectrophotometer. The expression level of associated HCMV transcripts was evaluated by reverse transcription-PCR (RT-PCR) using a PrimeScript RT-PCR Kit (Takara, Japan). The primer sets used for RT-PCR are listed in Table 1. The PCR products were separated on 1.5% agarose gels and visualized by ethidium bromide staining.

**Table 1:** Primers used for mRNA RT-PCR amplification of HCMV mRNAs.

Primer name	Nucleotide sequence (5′ to 3′)	Product size (bp)
UL123 forward	CAAGAG AAAGATGGACCCTG	242
UL123 reverse	CGAGTTCTGCCAGGACATC	
UL83 forward	TGCCCTGGATGCGATACTG	378
UL83 reverse	AGGACCTGACGATGACCCG	
LUNA round 1 forward	ATGACCTCTCCTCCACACC	556
LUNA round 1 reverse	GACGCTATATTTAGGGCTTCC	
LUNA round 2 forward	GAGCCTTGACGACTTGGTAC	241
LUNA round 2 reverse	GAGCCTTGACGACTTGGTAC	
GAPDH forward	GAGTCAACGGATTTGGTCGT	185
GAPDH reverse	GAGTCAACGGATTTGGTCGT	
hcmvIL-10 forward	GGGGAATTCATGCTGTCGGTGATGGTCT	381
hcmvIL-10 reverse	ACATTGCCGCATGTCTTTG	
LAcmvIL-10 forward	TGTTGAGGCGGTATCTGGAGA	420
LAcmvIL-10 reverse	CCGTCTTGAGTCCGGGATAG	

### Identification of transcription factor binding sites

The MATCH program, which is provided with the TRANSFAC (version 9.4) database (http://www.gene-regulation.com/cgi-bin/pub/programs/match/bin/match.cgi;Biobase Biological Databases), was used to identify putative transcription factor binding sites within the URR of the *UL111A* gene. The MATCH program [20] sets a point (0–1) for each base using a single nucleotide weighted model in TRANSFAC Professional 9.4 library [21].

### Chromatin immunoprecipitation (ChIP) assay and quantitative analysis of ChIP-PCR products

ChIP assays were carried out using an EZ ChIP Chromatin Immunoprecipitation Kit (Millipore, USA) according to the manufacturer’s recommendations. Briefly, the THP-1 cells (10^6^ cells/ml) infected with HCMV Towne strain (MOI = 5) for 4 days in the presence or absence of PMA, were cross-linked in 1% formaldehyde for 10 min at room temperature followed by glycine treatment for 5 min to quench unreacted formaldehyde. The cells were washed with PBS containing 5 μl 0.5% protease inhibitor cocktail and then lysed in 1 ml of SDS lysis buffer for 10 min on ice. The lysate was sonicated on ice to yield chromatin fragments of 200–1000 bp. Soluble chromatin was pre-cleared with 60 μl of Protein G agarose by rotation for 1 h at 4°C to remove proteins or DNA that may bind nonspecifically to Protein G agarose. Subsequently, the chromatin was incubated at 4°C overnight with the following primary antibodies: anti-AML-1 (Abcam, ab23980), anti-GATA1 (Abcam, ab11963), anti-C/EBP β (Abcam, ab32358), anti-histone H3 acetyl-K9 (Abcam, ab4441), anti-histone H3 dimethyl-K9 (Abcam, ab1220). The chromatin immunoprecipitates were harvested by adding 60 μl of Protein G agarose beads and washed sequentially with wash buffer. The cross-links of protein/DNA complexes were reversed by incubation in 5M NaCl at 65°C for 4–5 hours or overnight. Subsequently, the free DNA was purified using a DNA spin column. The immunoprecipitated DNA fractions were amplified by PCR with Ex Taq polymerase (Takara), and were used to detect the URR of HCMV *UL111A* gene. The primer sets used for ChIP-PCR are shown in Table 2. The PCR products were analyzed by 1.5% agarose gel electrophoresis, visualized by ethidium bromide staining, and quantified using Image J software.

**Table 2:** Primers used for ChIP-DNA PCR amplification.

Primer name	Nucleotide sequence (5′ to 3′)	Product size (bp)
AML-1 binding region forward	AAGCCGGGTTCGACCAAGA	200
AML-1 binding region reverse	GCGCAGTCACGGATAGGAT	
C/EBP β binding region forward	CGGTCATCATTCTGCTTCAC	225
C/EBP β binding region reverse	GGTTCCCTCTCTCTAATTCCC	
GATA-1 binding region forward	CTGATCCTATCCGTGACTGCG	250
GATA-1 binding region reverse	CCAAGCCTAGCTGCTCATTCG	

### Western blot analysis

The nucleoproteins from THP-1 cells were extracted using a Nuclear and Cytoplasmic Protein Extraction Kit (Beyotime) according to the manufacturer’s recommendations. Protein quantification was performed using a BCA Protein Assay Kit (Beyotime). Prior to western blot analysis, the extracted protein was diluted with sample buffer, and boiled for 10 min at 100°C. Protein samples (50 ug) were separated by sodium dodecyl sulfate-polyacrylamide gel electrophoresis (SDS-PAGE) and transferred to a nitrocellulose membrane. The blots were blocked in 5% skim milk in PBS containing 0.1% Tween-20 for 2 h at room temperature, and incubated with primary antibodies: anti-AML-1, anti-GATA, and anti-C/EBP β in dilution buffer (1:1000). Subsequently, the blots were washed, incubated with horseradish peroxidase-conjugated anti-mouse or anti-rabbit secondary antibodies (Beyotime), and detected using the beyoECL Plus (Beyotime). The signal intensity of indicated protein bands was quantified using the Image J software.

### Bisulfite Sequencing PCR (BSP)

Bisulfite modification method was used to analyze the methylation of the HCMV genomic DNA. Bisulfite treatment converts unmethylated cytosine residues (C) to uracil (U), which are converted to thymine (T) after PCR amplification, while methylated C is protected from conversion. Bisulfite-treated DNA was amplified by PCR, cloned and then sequenced. In our study, DNA denaturation and bisulfite conversion processes was integrated into one-step by a EpiTect Plus DNA Bisulfite Kit (QIAGEN) according to the manufacturer’s instructions. Bisulfite-treated genomic DNA was amplified by PCR using HS Taq polymerase (Takara) and primers spanning the URR of *UL111A* gene (from-4231 to-4001 bp and-4511 to-4149 bp relative to the *UL111A* gene). The primer sequences were:

URR1 forward primer: 5′-AGTAGAATAGAGATTTTTTGTT-3′; URR1 reverse primer: 5′-CTTCCACTTATTTTTTATTATT-3′.

URR2 forward primer: 5′-TGTTTGGTAAAGGAATAATT-3′; URR2 reverse primer: 5′-TCTCTAATTCCCTAAAAAACAAA-3′.

The PCR reaction mixtures were performed in a total of 25 μl containing 2.5μl of 10× PCR buffer, 2 μl of 25mM MgCl_2_, 2.5 μl of 25 mM deoxynucleotide triphosphates, 0.5 μl of each PCR primer (20 μM), 0.625 U of HS Taq (Takara) and 2 μl of the bisulfite modified DNA. The PCR amplification conditions were as follows: initial denaturation at 94°C for 5 min, followed by 35 cycles of 95°C for 30 s, annealing at 53.6°C (for the URR1 primer pair) or 43.3°C (for the URR2 primer pair) for 1 min, extension at 72°C for 1 min and finally 72°C for 10 min. The amplified fragment lengths were 212 bp (URR1 primer pair) and 362 bp (URR2 primer pair). PCR products were gel-purified by an Agarose Gel DNA Purification Kit (Takara) according to the manufacturer’s instructions. Purified PCR fragments were cloned into a pMD18-T simple vector (Takara), and the individual recombinant clones were sequenced to identify the presence of methylated CpGs within the URR of the *UL111A* gene. Sequencing of the bisulfite-modified DNA was performed by Invitrogen Biotechnology Co., Ltd. (USA), and the sequences of the PCR products were analyzed using DNAMAN software (Lynnon Biosoft, USA).

### RNA interference analysis

The lentivirus-mediated short hairpin RNA (shRNA) was designed for triggering of the gene silencing RNA interference (RNAi) pathway. For the transfection experiments, HCMV-infected THP-1 cells were seeded at 1×10^5^ cells/ml in RPMI-1640 medium before transfection, then HCMV-infected THP-1 cells were transfected with LV-shRNA-AML-1 (CCAGGTTGCAAGATTTAATGA) or negative control (LV-shRNA-NC: TTCTCCGAACGTGTCACGT) (GenePharma, Shanghai, China) according to manufacturer’s protocol. After 24 h post-transfection, half of the medium was changed, and the transfection efficiency of lentivirus-mediated shRNA into cells expressing GFP proteins was observed by FACS analysis at 72 h post-transfection. Total RNA and protein were extracted for further analysis.

## Results and Discussion

### HCMV genome maintenance and highly restricted transcription of viral genes in HCMV-infected THP-1 cells

**Fig 1:**
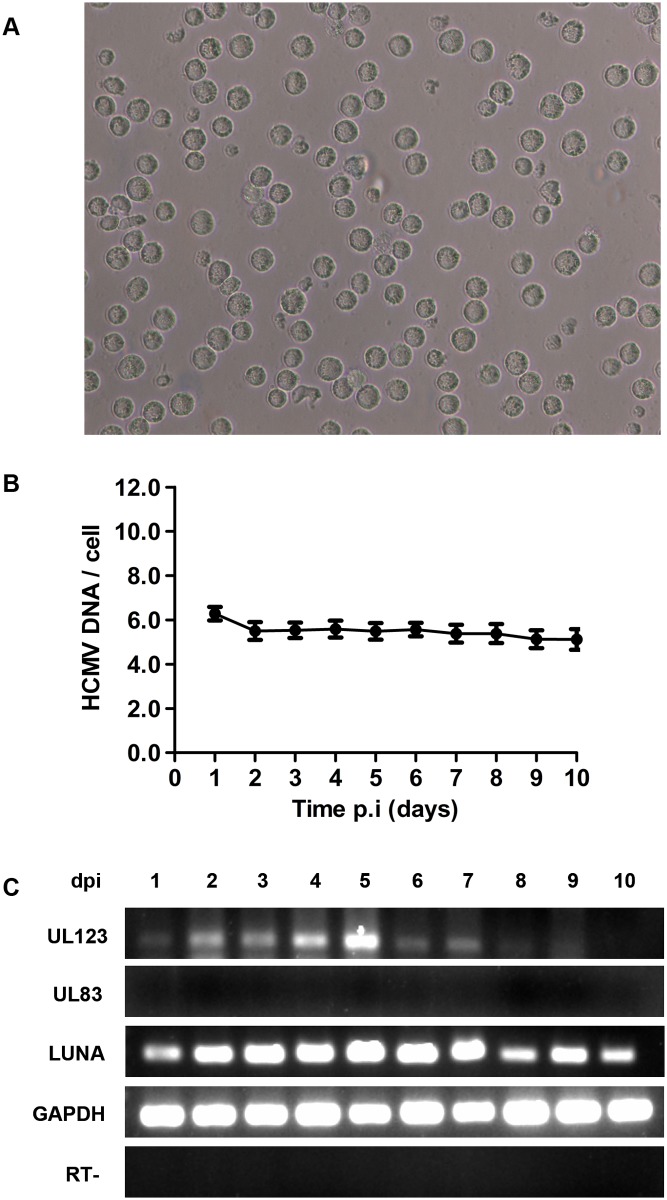
HCMV genome maintenance and transcription in infected THP-1 cells. (A) Microscopic examination of THP-1 cells infected with HCMV-Towne and grown in suspension (original magnification ×200). (B) Real-time qPCR analysis revealed the maintenance but lack of accumulation of the HCMV genome in infected THP-1 cells. Each sample was analyzed in triplicate. (C) Expression of HCMV transcripts (UL123, UL83, and LUNA mRNA) in infected THP-1 cells over a 10-day course of infection analyzed by RT-PCR. RT− represents RNA without prior reverse transcription, employed as a negative control. Dpi: day post infection.

We evaluated the maintenance and transcription of HCMV genome in infected THP-1 cells over a 10-day course of infection. Microscopic examination of the HCMV-infected THP-1 cells revealed a round morphology (Fig. 1A). Total DNA and RNA were isolated to quantify viral genome and transcripts, respectively. The viral IE1 gene was evaluated by qPCR to monitor viral DNA maintenance. The levels of UL123 (IE1), UL83 (pp65) and LUNA mRNA were determined using RT-PCR to assess viral gene transcription levels. In the infected THP-1 cells, HCMV DNA was maintained but failed to accumulate (Fig. 1B). The level of UL123 mRNA increased initially after infection, but decreased over time until the signal was undetectable; whereas the LUNA mRNA was detectable throughout the course of infection. In contrast, the expression of UL83 mRNA was undetectable over the 10-day course of infection (Fig. 1C). These findings are consistent with previous reports that in *ex vivo* primary cells, viral IE1 gene expression becomes activated at early stages of infection, and is subsequently repressed [22,23]. Collectively, our data suggest that the HCMV genome is maintained in HCMV-infected THP-1 cells, but its transcription prior to viral DNA replication is highly restricted.

### HCMV transcription in infected THP-1 cells following the addition of PMA

**Fig 2:**
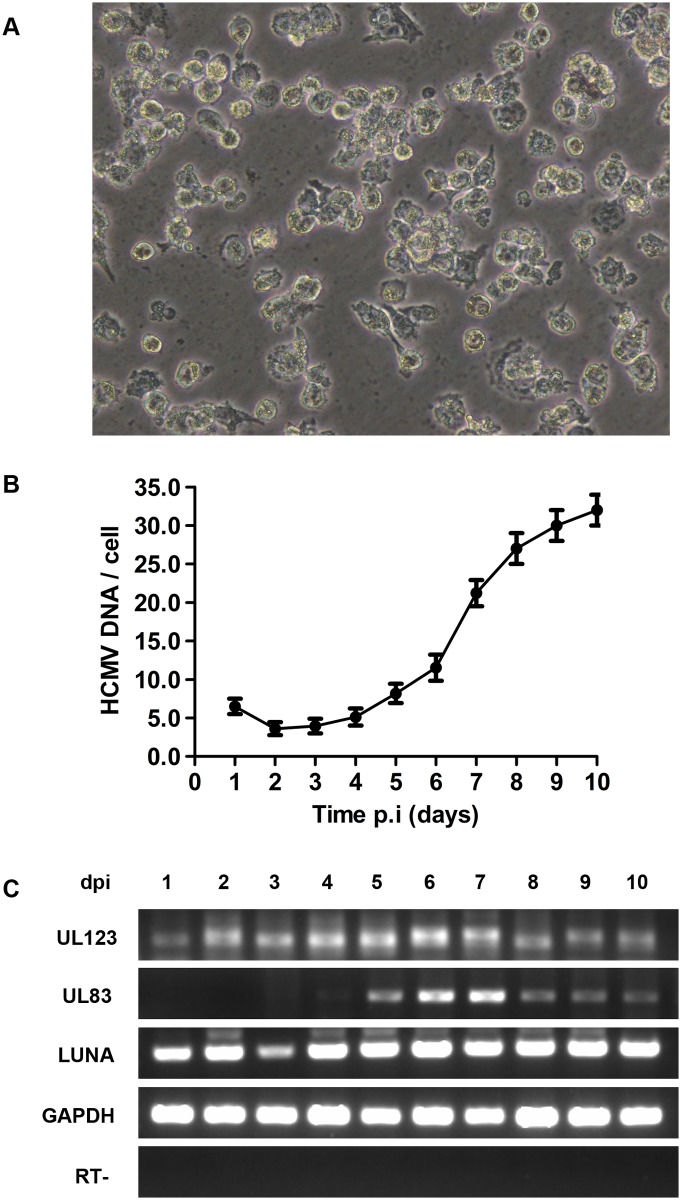
Induction of viral transcription in infected THP-1 cells with PMA treatment. (A) Microscope image showing differentiation of HCMV-infected THP-1 cells into an adherent and macrophage-like phenotype following stimulation with PMA (100 ng/μl) (original magnification ×200). (B) Viral DNA loads over time in HCMV-infected THP-1 cells following PMA treatment. Each sample was assayed in triplicate by qPCR. (C) Expression of viral transcripts in PMA-treated THP-1 cells infected with HCMV. RT− represents the RNA without prior reverse transcription, employed as a negative control.

PMA is commonly used to reactivate viral transcription in *ex vivo* herpesvirus latency model systems, including Epstein-Barr virus (EBV) [24] and Kaposi’s sarcoma-associated herpesvirus (KSHV) [25]. In this study, stimulation of HCMV- infected THP-1 cells with PMA resulted in differentiation of the cells into an adherent macrophage-like phenotype (Fig. 2A). Macrophages have been shown to be important in HCMV reactivation [26–28]. In fact, partial HCMV reactivation has been observed in THP-1 [29], and NTera2 [30] cell lines after treatment with PMA. Therefore, we next investigated whether the HCMV genes were transcribed in THP-1 cells following PMA stimulation. To achieve this, DNA and total RNA were extracted over 10 days from HCMV-infected cells cultured in the presence of PMA, and the replication of HCMV DNA and accumulation of UL123, UL83 and LUNA mRNAs were monitored as described above. As shown in Fig. 2B, the viral DNA loads started to increase at day 5 following PMA treatment, suggesting that the infection of differentiated THP-1 cells with the Towne strain of HCMV is productive. Notably, the UL123 mRNA level in the differentiated THP-1 cells gradually increased over time, and rapidly reached its *peak* at day 5. The expression of UL83 mRNA was detected at about day 3 post-induction with PMA and continued to increase thereafter. The expression of LUNA mRNA in the differentiated THP-1 cells was observed throughout the course of infection (Fig. 2C). Our results show that HCMV gene transcription is more abundant in THP-1 cells treated with PMA than untreated cells, suggesting that cell differentiation promotes HCMV reactivation in PMA-treated THP-1 cells.

### Upregulation of hcmvIL-10 expression in differentiated THP-1 cells

**Fig 3:**
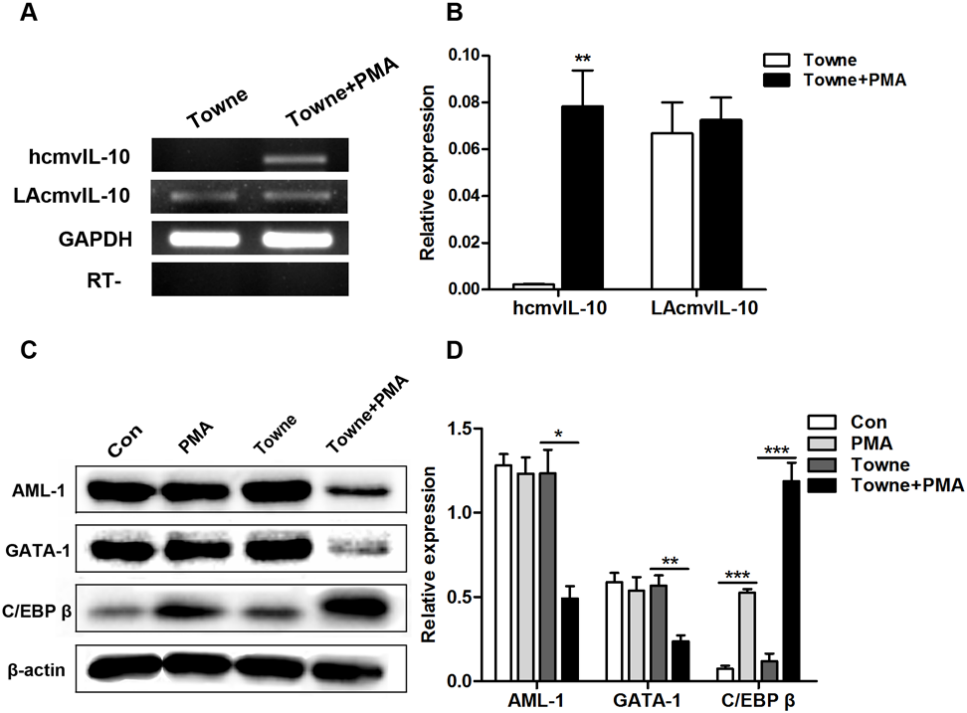
Transcription of the *UL111A* gene and expression of transcription factors in PMA-treated and untreated THP-1 cells infected with HCMV. (A) Expression levels of the hcmvIL-10 and LAcmvIL-10 transcripts in differentiated and undifferentiated HCMV-infected THP-1 cells, analyzed by RT-PCR. (B) Quantitative analysis of the data in (A) from three independent experiments. RNA transcripts were normalized to cellular GAPDH. (C) Western blots showing the expression level of AML-1, GATA-1, and C/EBP β proteins in differentiated and undifferentiated HCMV-infected THP-1 cells. (D) Quantitative analysis of the data in (C) from three independent experiments. Proteins were normalized to cellular β-actin. *P < 0.05, **P < 0.01, ***P < 0.001.

HcmvIL-10, a functional homolog of hIL-10, is transcribed from the HCMV *UL*111A gene during productive infection [12,31]. However, the expression of a similar transcript, the latency-associated cmvIL-10 (LAcmvIL-10) from the alternative splicing of the *UL*111A region has been reported both during latent infection [32] and productive infection [33], suggesting that LAcmvIL-10 is latency-associated rather than latency-specific [13]. To determine whether hcmvIL-10 was expressed in HCMV-infected THP-1 cells treated with PMA, RT-PCR was used to analyze total RNA isolated from infected cells cultured for 4 days in the presence or absence of PMA. Expectedly, the expression of hcmvIL-10 mRNA was undetectable in untreated THP-1 cells, whereas the expression of hcmvIL-10 mRNA was significantly increased in PMA-treated differentiated THP-1 cells (Fig. 3A, 3B). However, the expression of LAcmvIL-10 can be detected both in PMA untreated and treated infected THP-1 cells. These data suggest that macrophage differentiation of HCMV-infected THP-1 cells induced by PMA treatment may upregulate hcmvIL-10 expression.

Taken together, it is evident from our above findings that HCMV infection is not productive in untreated THP-1 cells. Monocytes differentiation is required for being permissive to HCMV replication [34]. The detection of hcmvIL-10 transcription in differentiated cells, but not in undifferentiated THP-1 cells, further suggests that hcmvIL-10 transcription might be facilitated by myeloid differentiation.

### Expression of transcription factors in HCMV-infected THP-1 cells

We next sought to examine the changes in the expression levels of the transcription factors AML-1, GATA-1 and C/EBP β in PMA-treated and untreated HCMV-infected THP-1 cells. For this purpose, nucleoproteins were extracted from the infected cells at 4 dpi, and subjected to western blot analysis using specific antibodies. Strikingly, in uninfected cells, AML-1 and GATA-1 expression was similar in the PMA-treated and untreated cells, whereas in HCMV-infected cells, AML-1 and GATA-1 expression was much lower in PMA-treated cells than in untreated cells. Conversely, the expression of C/EBP β was significantly higher in PMA-treated cells compared to untreated cells (Fig. 3C, 3D). Therefore, our results indicate that a differential expression of transcription factors occurs in different phases of cell differentiation and development.

### Identification of functional transcription factor binding sites in the *UL111A* gene promoter

**Fig 4:**
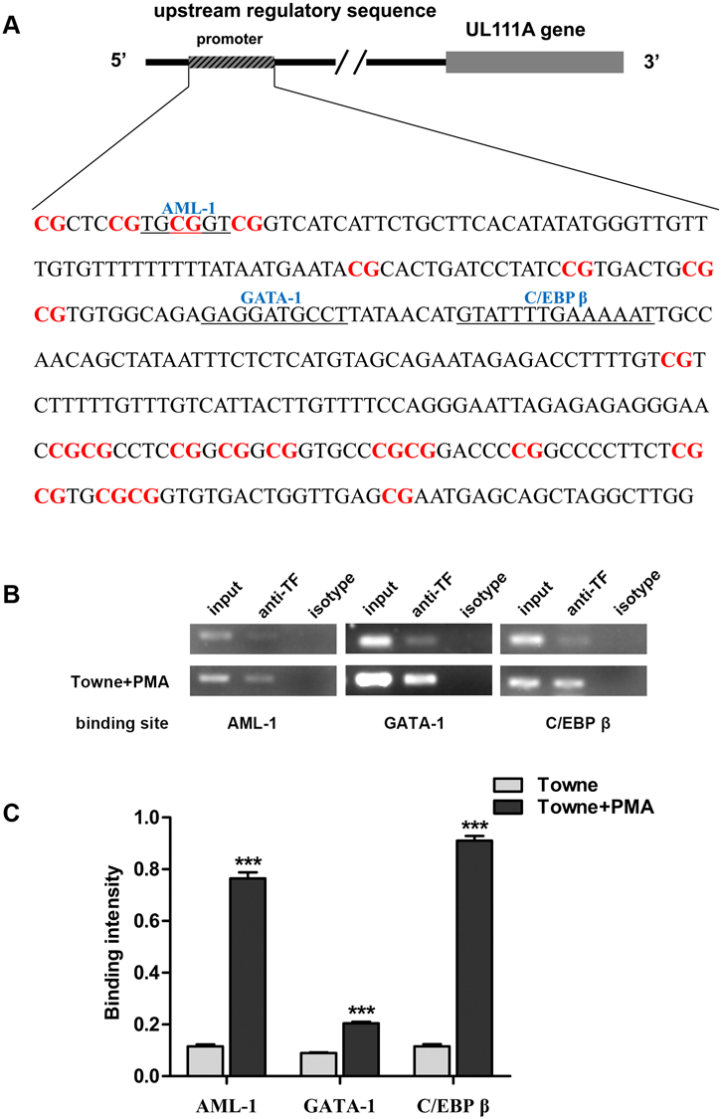
Identification of transcription factor binding sites in the URR of the *UL111A* gene. (A) Potential binding sites for the myeloid transcription factors AML-1, C/EBP β, and GATA-1 in the URR of the *UL111A* gene predicted using the MATCH program and TRANSFAC database. (B) ChIP analysis of three transcription factors binding to their corresponding sites. The lanes are designated as: “input”—PCR amplification of input DNA, “anti-TF”—PCR amplification of chromatin DNA fragments precipitated by antibodies against transcription factors, “isotype”—a control for non-specific reactions. (C) Comparison of transcription factor binding between undifferentiated and differentiated THP-1 cells. Quantitative analysis of the data is from three independent experiments. *** *P* < 0.001.

In order to identify the transcription factors that may be responsible for regulating *UL111A* gene function during HCMV infection, we initially identified putative transcription factor binding sites in the URR of the *UL111A* gene by bioinformatics analysis. Notably, three adjacent putative transcription factor binding consensus sequences (AML-1, GATA-1 and C/EBP β) were identified in the URR of *UL111A* (Fig. 4A). We then conducted quantitative analyses to determine the binding of these three transcription factors to the *UL111A* URR using ChIP assays with antibodies against each transcription factor. The binding intensities of AML-1, GATA-1 and C/EBP β to the *UL111A* URR were found to be significantly higher in PMA-treated THP-1 cells than in untreated cells (*P* <0.05; Fig. 4B, 4C).

Collectively, our results indicate that the three myeloid transcription factors, AML-1, GATA-1 and C/EBP β are recruited efficiently to the *UL111A* gene promoter upon PMA-induced differentiation in HCMV-infected THP-1 cells, and the recruit is independent of the expression level of the transcription factors.

### Differential pattern of histone modification in the URR of *UL111A* in differentiated and undifferentiated THP-1 cells

**Fig 5:**
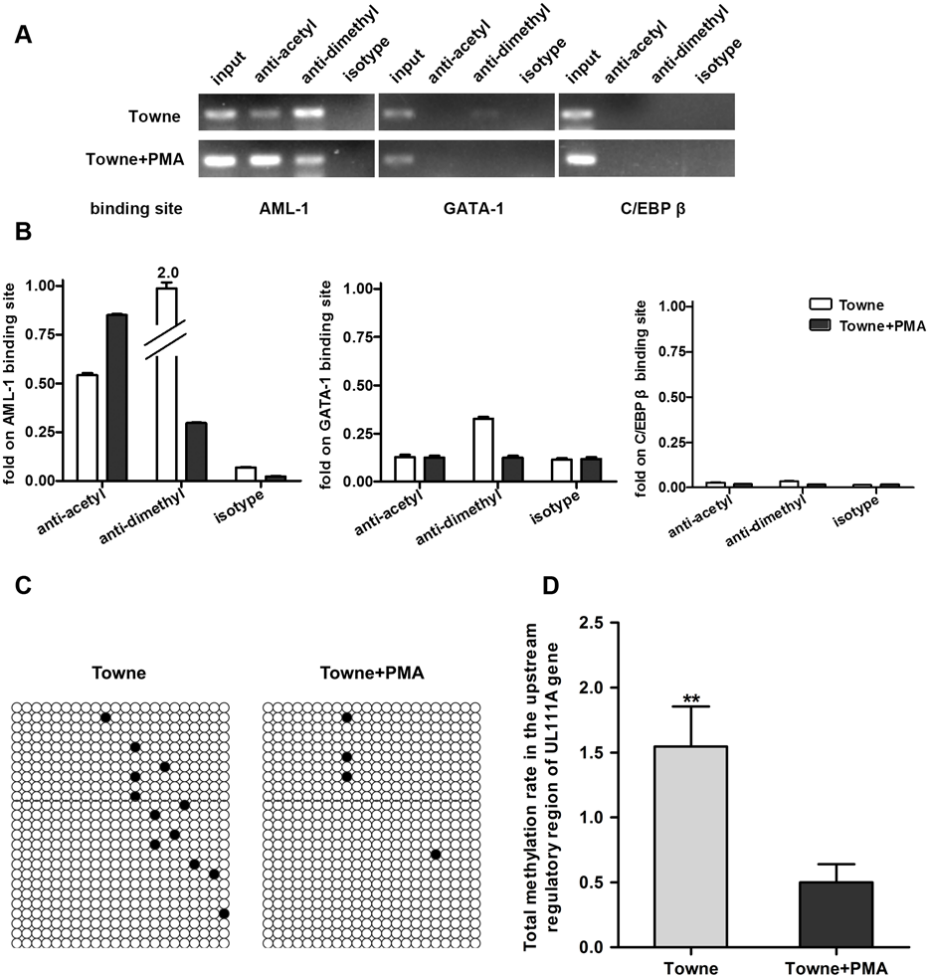
Epigenetic modification status in the URR of *UL111A* from undifferentiated and differentiated HCMV-infected THP-1 cells. (A) ChIP analysis showing the association of the transcription factors binding sites with histone H3 acetylated at K9 position (H3 acetyl-K9) and dimethylated at K9 position (H3 dimethyl-K9). The lanes are designated as: “input”—PCR amplification of input DNA, “anti-acetyl”—PCR amplification of the chromatin DNA fragments precipitated by antibody against H3 acetyl-K9, “anti-dimethyl”—PCR-amplification of the chromatin DNA fragments precipitated by antibody against H3 dimethyl-K9, “isotype”—a negative control for non-specific reactions. (B) Quantitative analysis of the data in (A) from three independent ChIP experiments. The results were normalized to the input signal, which was set to 1. (C) Methylation status of the CpG sites in the URR of the *UL111A* gene in undifferentiated and differentiated infected THP-1 cells infected with HCMV. The methylation status of individual CpG dinucleotides is indicated as: ○ and ●, which represent unmethylated and methylated cytosine, respectively. (D) Quantitative analysis of the total methylation data in (C) from three independent BSP experiments. **P < 0.01.

Recently, it has been reported that histone modifications at the epigenetic level are associated with the transcriptional regulation of various herpesvirus genomes [35–38]. Histone posttranslational modifications are known to control gene expression. It is widely believed that methylation of histone H3 at the K9 position is usually associated with transcriptional repression, whereas acetylation at this position enhances gene transcription [39]. Therefore, we hypothesize that histone H3 K9 modifications may play an important role in the regulation of hcmvIL-10 mRNA expression. To test this hypothesis, a ChIP assay was used to examine the presence of histone H3 modifications in the transcription factor (AML-1, GATA-1 and C/EBP β) binding sites of *UL111A* URR with antibodies against H3 acetyl-K 9 or H3 dimethyl-K9. Stark differences in histone H3 modifications were observed between differentiated and undifferentiated THP-1 cells infected with HCMV (Fig. 5A, 5B). In the undifferentiated cells, the AML-1 and GATA-1 binding sites were predominantly associated with H3 dimethyl-K9 modification, while the C/EBP β binding site showed no histone H3 K9 modifications. However, in the differentiated cells, the AML-1 binding site was associated with a H3 acetyl-K9 modification, while the C/EBP β and GATA-1 binding sites showed no histone H3 K9 modifications. These results are consistent with previous studies, which showed that HCMV modulates the chromatin modification machinery of the host cellular system to control the expression of viral genes in both undifferentiated and differentiated monocytes [40–42]. Hence, our findings suggest that, histone H3 modifications of the AML-1 binding site may influence the activation of the *UL111A* gene.

### Analysis of DNA methylation status in the URR of *UL111A* in PMA-treated and untreated THP-1 cells

To investigate whether DNA methylation occurs in the *UL111A* URR, methylation of the CpG dinucleotides in the *UL111A* URR (spanning from-4511 to-4001 bp relative to the *UL111A* gene) was analyzed using the bisulfite modification method. The DNA methylation status of the binding sites for AML-1, C/EBP β and GATA-1 was analyzed in HCMV genomic DNA extracted from undifferentiated and differentiated THP-1 cells at 4 dpi. Surprisingly, no significant difference was observed in the methylation status of each binding site between PMA treated and untreated cells (P > 0.05). However, there was a significant difference in the total methylation status of the region containing all three transcription factor binding sites (χ^2^ = 5.18, *P* < 0.05) (Fig. 5C, 5D). These results suggest that hcmvIL-10 expression is not directly regulated by the DNA methylation of a specific transcription factor binding site, rather the total methylation status of the entire URR may be involved in such regulation.

AML-1 belongs to the CBF family of transcription factors, whose members are heterodimers consisting of a α-subunit (CBF α), which binds to the DNA consensus sequence TGT/CGGT, and a β-subunit (CBF β) that enhances the DNA-binding affinity through α-subunit rather than binding to DNA directly [18,19,43]. Interestingly, it has been reported that AML-1 cooperates synergistically with PU.1 and C/EBP proteins to regulate monocyte/macrophage differentiation in monocytic cells [44,45]. However, further investigation is needed to determine whether the three adjacent AML-1, GATA-1 and C/EBP β binding sites identified in the present study cooperate to regulate hcmvIL-10 expression.

### AML1 suppression affects the *UL111A* gene expression

**Fig 6:**
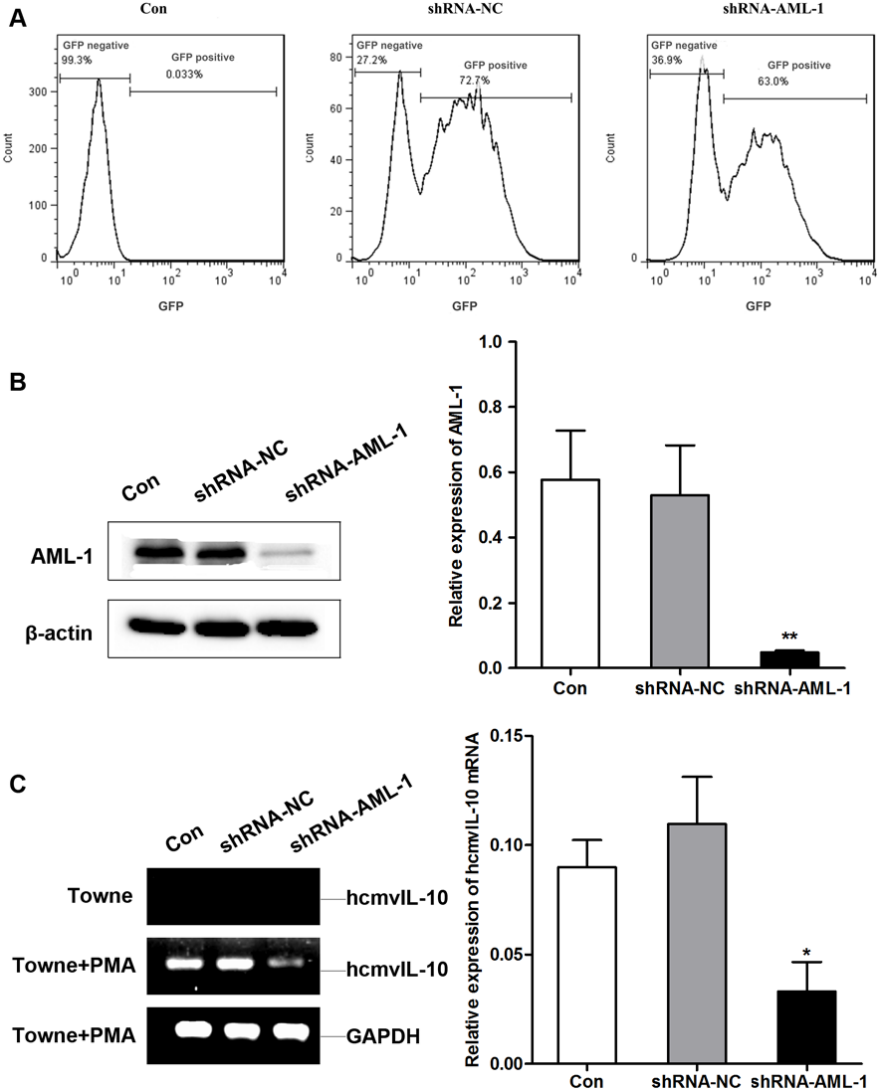
AML-1 suppression affects the *UL111A* gene expression. (A) FACS analysis of transfection efficiency. The infection efficiency was measured by FACS analysis in HCMV-infected THP-1 cells transfected with LV-shRNA-AML1 or LV-shRNA-NC at 72 h post-transfection. (B) Western blot was detected in HCMV-infected THP-1 cells transfected with LV-shRNA-AML1 or LV-shRNA-NC. **P<0.01 Verse LV-shRNA-NC. (C) HCMV-infected THP-1 cells stimulated with PMA were transfected with LV-shRNA-AML1 or LV-shRNA-NC for 72h. RT-PCR was taken to analyze the change of hcmvIL-10. *P<0.05. Verse LV-shRNA-NC.

We investigated whether silencing of endogenously expressed AML-1 led to a decrease in the *UL111A* gene expression in HCMV-infected THP-1 cells. We designed a shRNA, LV-shRNA-AML-1, which targets the mRNA of human AML-1. The expressions of AML-1 protein and cmvIL-10 mRNA were detected in HCMV-infected THP-1 cells transfected with LV-shRNA-AML-1 or LV-shRNA-NC by western blot and RT-PCR, respectively. As revealed in Fig. 6A, almost 70% cells harbored GFP fluorescence 72 h after transfection, as detected by FACS. Expectedly, the transfection of LV-shRNA-AML-1 significantly decreased the endogenous AML-1 in these cells (Fig. 6B). Introduction of LV-shRNA-AML-1 also diminished about 70% amount of hcmvIL-10 mRNA compared with control shRNA (Fig. 6C). These results firmly convinced that AML-1 contributes to the enhanced expression of the *UL111A* gene by binding to the UUR.

## Conclusions

Our study presents the first evidence that the myeloid transcription factor, AML-1 may contribute to the epigenetic activation of the HCMV *UL111A* gene via histone modification in differentiated THP-1 cells. Based on our results and given the influence of hcmvIL-10 on macrophage polarization, we propose a working model for the contribution of AML-1-expressing myeloid cells to immune evasion in HCMV infection. We speculate that HCMV requires the differentiation of myeloid cells to sustain its replication, and utilizes myeloid transcription factors, such as AML-1, to activate the expression of the immune mediator homolog hcmvIL-10 to maintain immune evasion. Detailed future studies will aim at elucidating the precise mechanism underlying this process.
